# Efficacy of radial incision combined with tunnel floating line drainage in the treatment of high posterior horseshoe anal fistula and perianal flora: Randomized control trial

**DOI:** 10.1097/MD.0000000000039947

**Published:** 2024-10-11

**Authors:** Hang Yi, Yong Zheng, Zhengqing Yan

**Affiliations:** a Huazhong Agricultural University Hospital, Wuhan, Hubei, P. R. China; b Department of Anorectal Surgery, Wuhan Hospital of Traditional Chinese Medicine, Wuhan, Hubei, P. R. China; c Department of Surgery, Wuhan University of Technology Hospital, Wuhan, Hubei, P. R. China.

**Keywords:** anal fistula, anal function, arc incision internal drainage, perianal flora, radial incision

## Abstract

**Background:**

Due to the high prevalence of posterior horseshoe anal fistula and causing numerous complications, this study aimed to investigate the clinical effect of radial incision combined with tunnel floating line drainage (RCTD) and arc incision internal drainage in the treatment of the disease and the influence on perianal flora.

**Methods:**

Ninety-six subjects treated with high posterior horseshoe anal fistula were stochastically assigned to a joint group (RCTD), and control group (arc incision internal drainage). The operation-related conditions, complication rate, anal function, and recurrence rate of 6 months after operation were compared, and perianal secretions were collected before operation and 1 day after operation to detect the changes of microbial flora.

**Results:**

After operation, it was corroborated notable difference between joint group and control group in operation time, intraoperative blood loss, wound healing time, visual analogue scale score 6 hours after operation and phase I cure rate. Chi square test analysis showed notable difference between control group (27.08%) and joint group (10.40%) in incidence of complications, in terms of number of pathogens detected around anus, significantly smaller of the incremental change for the joint subgroup versus the control subgroup 1 day after operation.

**Conclusion:**

RCTD can be the best choice for patients with high posterior horseshoe anal fistula. This operation method has the advantages of short operation time, less trauma, fewer complications, fast recovery of anal function, and can also reduce perianal pathogenic bacteria infection.

## 1. Introduction

Anal fistula is a common disease in anorectal department. The main cause is that the rectum and anal canal are invaded by inflammation, which causes perianal tissue to fester and form a fistula. If the fester invades the part above the sphincter, it is called high anal fistula.^[1,2]^ High posterior horseshoe anal fistula refers to the horseshoe anal fistula with the inner mouth at the back, which is also the most common type.^[3]^ According to the survey,^[4]^ the recurrence rate of high posterior horseshoe anal fistula is high, and it is easy to lead to anal incontinence, so it should be treated in time. The treatment principle of anal fistula is to ensure the sphincter function while curing. Arc incision internal drainage is a classic operation for anal fistula, but the high posterior horseshoe anal fistula has complex shape, obvious trauma during operation, difficult suture, and limited application value.^[5,6]^ Some scholars found that making radial incision at both ends of the fistula branch and then giving tunnel floating line drainage can improve the cure rate of high anal fistula, reduce sphincter damage and protect anal function.^[7]^ This method involves creating a radial incision to expose the fistula tract, followed by carefully inserting a seton to facilitate drainage and healing. The report shows that the infection of the internal mouth of anal fistula forms the external mouth, and the pathogen invades the pipeline from the external mouth, causing the tissue to collapse, forming a complex anal fistula. The perianal pathogen infection is one of the important reasons that lead to the disease progress and affect the surgical prognosis.^[8]^ Radial incision combined with tunnel floating line drainage (RCTD) leverages the principle of minimizing tissue disruption while ensuring adequate drainage of the fistula tract. By employing a radial incision, the technique allows for precise access to the fistula with minimal damage to surrounding tissues.^[9]^ The subsequent placement of a tunnel floating line encourages gradual drainage and healing, reducing the potential for abscess formation and infection. This targeted approach not only promotes faster recovery but also significantly lowers the risk of postoperative complications, a pivotal consideration in the management of horseshoe anal fistula. The benefits of RCTD extend beyond the immediate postoperative period, contributing to improved long-term outcomes for patients.^[10]^ The technique’s emphasis on tissue preservation is particularly advantageous, reducing the likelihood of incontinence and other sphincter-related complications commonly associated with traditional fistula surgery. Moreover, the strategic use of tunnel floating line drainage minimizes the chances of fistula recurrence, a frequent concern in fistula treatment.^[11]^ RCTD offers a compelling blend of efficacy, safety, and patient quality of life through these advantages. In the context of preventing fistula complications such as infection, the RCTD technique stands out for its proactive approach. Ensuring comprehensive drainage and facilitating a controlled healing environment, significantly reduces the environment conducive to bacterial growth and infection.^[12]^ This preventative strategy is crucial in managing fistulas, where the risk of infection can complicate recovery and exacerbate patient discomfort. Thus, the integration of radial incision with tunnel floating line drainage in the treatment of high posterior horseshoe anal fistula represents a significant advancement, offering a refined surgical strategy that addresses the complexities of the condition while prioritizing patient safety and recovery. In view of this, this study compared the therapeutic effects of the 2 methods mentioned above, as well as the changes of perianal flora, to provide reference for the surgical selection of patients with high posterior horseshoe anal fistula.

## 2. Methods and materials

### 2.1. Design and procedures

This investigation was conducted as a randomized controlled trial with a double-arm design and repeated measurements from October 2021 to October 2022. The trial aimed to evaluate and compare the effectiveness of the RCTD technique against the arc incision internal drainage (AID) method. Specific outcomes of interest included improvements in operation-related conditions, complication rates, anal function, recurrence rates 6 months postoperation, and changes in microbial flora. Assessments were carried out at 2 points: baseline and after the surgery. The AID method was applied to the control group, while the RCTD technique was utilized for the experimental subgroup.

### 2.2. Inclusion and exclusion criteria

The selection and disqualification of participants were assessed based on predefined criteria. Following evaluation, eligible participants were randomly allocated to the study groups. The inclusion criteria included: (1) individuals aged between 18 and 60 years; (2) patients meeting the diagnostic standards for high posterior horseshoe anal fistula as referenced^[13]^; (3) all participants undergoing surgical intervention; and (4) availability of complete clinical data. The exclusion criteria were: (1) patients with anal fistula types other than the specified; (2) individuals suffering from severe systemic diseases; (3) those with acute or chronic infections, tuberculosis, Crohn disease, and similar conditions; (4) patients diagnosed with malignant tumors; and (5) women who were pregnant or breastfeeding. Withdrawal criteria included: (1) participants who did not complete the study; and (2) participants who were lost to follow-up. No participants were withdrawn from the study. The ethics committee of the Wuhan University of Technology Hospital granted approval for this research.

### 2.3. Preoperative preparation

Before surgery, all necessary diagnostic tests were completed. On the day of surgery, patients were required to fast, abstain from drinking water, and undergo gastrointestinal cleansing. Following intraspinal anesthesia, subjects from both groups were positioned on the operating table, where standard sterilization procedures were implemented, and sterile drapes were placed.

### 2.4. Intervention

**Figure 1.**
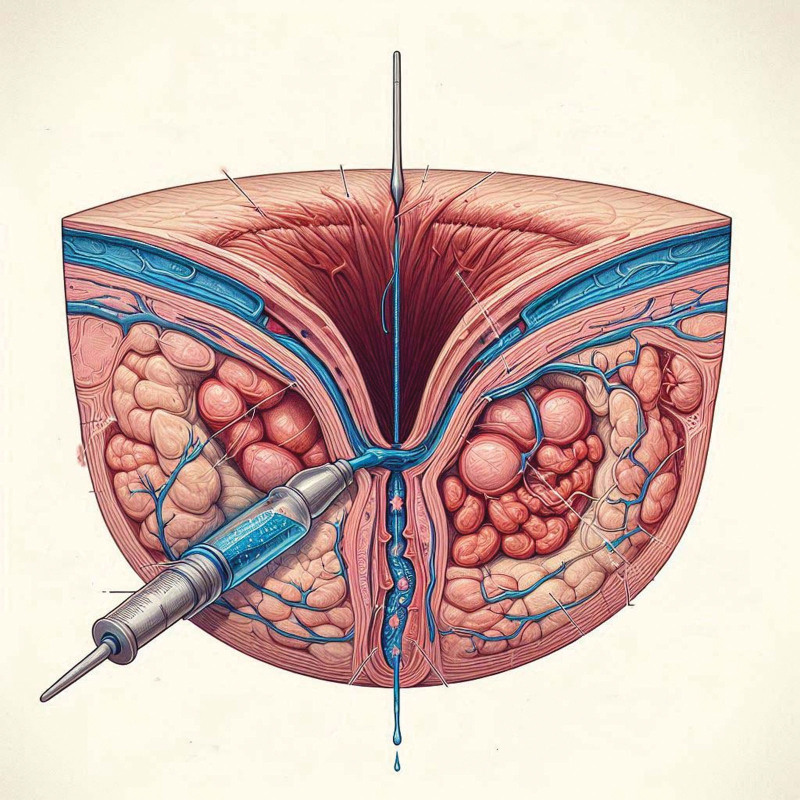
Symbolic design of intervention steps: (1) identification of fistula tract: shows the use of a probe and methylene blue staining; (2) radial incision combed: demonstrates the radial incision at the posterior midline; (3) incision and tissue removal: illustrates the “V”-shaped incision with necrotic tissue removal; (4) tunnel floating line drainage: shows the placement of a seton and irrigation of the lumen.

In the experimental group, the RCTD technique was executed. Initially, the direction of the fistula was identified using methylene blue staining. A probe was then inserted through the fistula’s external opening, guiding a radial incision made at the anus’s posterior midline down to the depth of the fistula probe. This allowed for the determination of the pathogen’s trajectory. The internal opening of the anal fistula was incised, and both the internal sphincter below the dentate line and the superficial part of the external sphincter were sequentially incised, extending 1 to 2 cm towards the anal margin. Contaminated and necrotic tissue within the anal canal was removed with a curette, and the incision was shaped into a “V” for optimal drainage. Subsequently, tunnel floating line drainage was performed; the internal opening was radially excised, the posterior incision’s fistula wall was scraped and cleaned, the lumen was irrigated, and a rubber strip was placed between the 2 incisions to adjust the tightness. In cases of an excessively long fistula, an additional mid-incision could be made for enhanced drainage. Bleeding was controlled, and the wound was dressed. Talking about perianal flora, it is important to note that all patients included in the study received prophylactic antibiotics before the operation. The same antibiotic regimen was used for both groups, with ceftriaxone administered as the antibiotic of choice for induction. This was done to reduce the risk of infection during surgery and to ensure uniformity in the treatment across both groups. Postoperative care included prophylactic antibiotics, monitoring of incision changes and drainage fluid characteristics, and regular gauze changes. The control group received the AID treatment. Preoperative preparations, fistula exploration, and treatment of the internal opening were conducted in the same manner as in the experimental group (Fig. 1).

### 2.5. Data collection

#### 2.5.1. Operation-related situation

The data collected included the duration of the surgery, the volume of blood lost intraoperatively, and the Phase I cure rate. The Phase I cure rate encompasses the absence of clinical symptoms, complete healing of the anal fistula and wound, no requirement for a secondary drainage procedure, and no recurrence within a six-month period. Additionally, wound healing time and the visual analogue scale (VAS) score, recorded 6 hours post-surgery, were also measured.^[14]^ The VAS score, ranging from 0 to 10 points, is utilized to assess the patient’s level of pain, with higher scores indicating greater pain intensity.

#### 2.5.2. Assessment of complications

The postoperative complications of the 2 groups were statistically analyzed, including postoperative bleeding, urinary retention, anal deformity, anal incontinence, infection, etc.

#### 2.5.3. Assessment of anal function

Wexner anal incontinence score^[15]^ was used to evaluate the anal function of patients in both groups before and 1 month after the operation. The score includes 5 aspects: control of gas, control of liquid stool, control of solid stool, the need for pads, and changes in lifestyle. For each item, a score ranging from 0 to 4 points was given (None = 0, Occasionally = 1, Sometimes = 2, Often = 3, Always = 4), with a total possible score of 20. The lower the score, the better the anal function. In this study, the aspects of control for liquid and solid stool were combined into 1 item for clarity.

#### 2.5.4. Assessment of recurrence rate

(1) The external mouth and wound of the anal fistula had not healed after 1 month. (2) New external orifice of anal fistula was discovered. (3) Local anal fistula symptoms occurred for the second time. Any of the above 3 items can be diagnosed by combining imaging examination.^[16]^

#### 2.5.5. Assessment changes of perianal microbial flora

The perianal secretion samples of the subjects were collected before operation and 1 day after operation for bacterial culture. There were colonies growing in the culture dish was positive, but no colony was negative after 48 hours. At the same time, the bacterial strains were identified by automatic microbiological analyzer.

### 2.6. Data statistics

The data are analyzed by SPSS 24.0. The measurement data were in accordance with normal distribution and the variance was described by mean and SD, and *t* value test was performed. The counting data were described by (n [%]) table, and tested by χ^2^ value/ Fisher exact probability method. When *P* < .05, it was considered that the difference was statistically significant.

## 3. Results

### 3.1. The effect of RCTD on operation-related situation and complication situation

**Figure 2.**
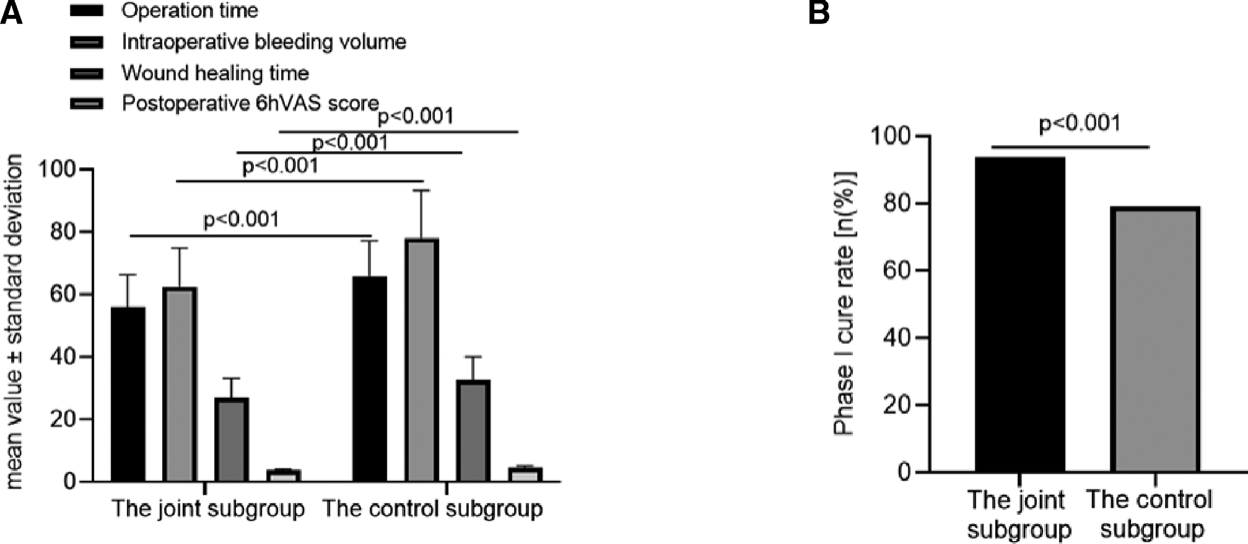
(A) and (B) Basic data related to the participants’ surgery.

**Figure 3.**
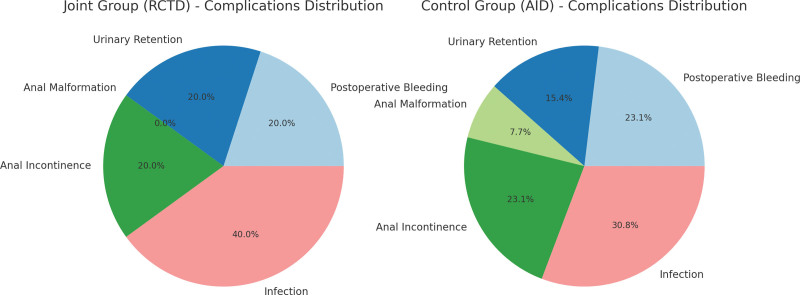
Descriptive data related complications of disease.

Compared to the control group, the joint group experienced a reduction in operation duration, wound healing time, and intraoperative blood loss. Additionally, it exhibited an increased Phase I cure rate and a decreased postoperative 6-hour VAS score, as evidenced in Table 1 and Figure 2. Regarding the occurrence of complications, the joint subgroup reported 5 instances, resulting in a 10.42% incidence rate, whereas the control subgroup encountered 13 instances, equating to a 27.08% incidence rate. The frequency of complications in the joint subgroup was significantly lower when compared to the control subgroup, as detailed in Table 2 and Figure 3.

**Table 1:** Basic data related to the participants’ surgery.

Groups	N	Operation time (min)	Intraoperative bleeding volume (mL)	Phase I cure rate	Wound healing time (days)	Wound healing time	Postoperative 6 hours VAS score
The joint group	48	56.02 ± 10.39	62.58 ± 12.40	45 (93.75)	27.15 ± 6.08	27.15 ± 6.08	3.68 ± 0.55
The control group	48	65.87 ± 11.35	78.14 ± 15.23	38 (79.17)	32.62 ± 7.54	32.62 ± 7.54	4.57 ± 0.64
χ^2^/*t*		4.435	5.489	4.360*	3.913	3.913	7.307
*P*		<.001	<.001	<.001	<.001	<.001	<.001

VAS = visual analogue scale.

*Note*: Data were expressed as mean ± SD or frequency (%).

* Marked with χ^2^ value.

**Table 2:** Descriptive data related complications of disease.

Grouping	N	Postoperative bleeding	Urinary retention	Anal malformation	Anal incontinence	Infected	Total occurrence
The joint group	48	1 (2.08)	1 (2.08)	0 (0.00)	1 (2.08)	2 (4.16)	5 (10.40)
The control group	48	3 (6.25)	2 (4.17)	1 (2.08)	3 (6.25)	4 (8.33)	13 (27.08)
χ^2^							4.376
*P*							.036

*Note*: Data were expressed as frequency (%).

### 3.2. The effect of RCTD on anal function and recurrence rate

**Figure 4.**
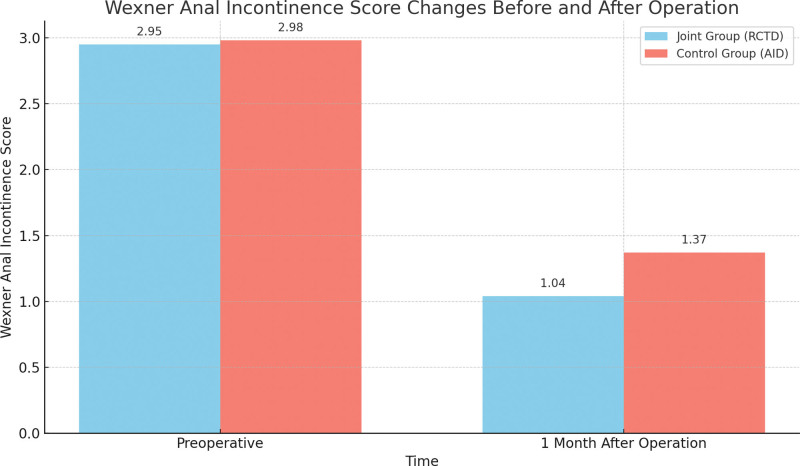
Wexner anal incontinence score changes in joint and control groups before and 1 month after the operation.

One-month postoperation, the Wexner scores for anal incontinence were significantly reduced in both subgroups when compared to the preoperative scores, with a more pronounced decrease observed in the joint subgroup, as presented in Table 3 and Figure 4. A 6-month follow-up revealed no instances of recurrence in the joint subgroup (0.00%) and 1 instance of recurrence in the control subgroup (2.08%). There was no statistically significant difference in the rates of postoperative recurrence between the 2 groups, as determined by the Fisher exact probability method (*P* = 1.000).

**Table 3:** Wexner anal incontinence score changes in joint and control groups before and 1 month after the operation.

Grouping	N	Preoperative	1 month after operation	*t*	*P*
The joint group	48	2.95 ± 0.64	1.04 ± 0.35	18.141	<.001
The control group	48	2.98 ± 0.62	1.37 ± 0.41	15.007	<.001
*t*		0.233	4.241		
*P*		.816	<.001		

### 3.3. The effect of RCTD changes of perianal microbial flora

**Figure 5.**
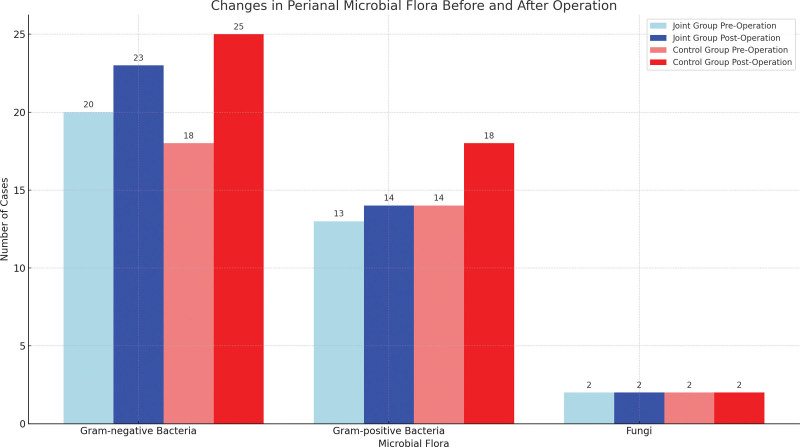
Perianal microbial flora (plant) changes in joint and control groups before and after the operation.

Prior to surgery, 35 strains of pathogens were identified around the anus in the joint subgroup, compared to 34 strains in the control subgroup. The predominant perianal pathogens identified in both subgroups were gram-negative bacteria, with *Escherichia coli*, *Klebsiella pneumoniae*, and *Proteus mirabilis* being the most prevalent. Following surgery, an additional 4 strains of pathogens were detected in the joint subgroup, whereas the control subgroup saw an increase of 11 strains. This difference in the increment of pathogens between the 2 subgroups was statistically significant, as shown in Table 4 and Figure 5.

**Table 4:** Perianal microbial flora (plant) changes in joint and control groups before and after the operation.

Microbial flora	The joint subgroup (n = 48)			The control subgroup (n = 48)		
	Preoperative	Postoperative	Newly added	Preoperative	Postoperative	Newly added
Gram-negative bacteria	20	23	3	18	25	7
*Escherichia coli*	6	5		7	6	
*Klebsiella pneumoniae*	5	6		4	5	
*Proteus mirabilis*	4	4		4	4	
Others	5	8		3	10	
Gram-positive bacteria	13	14	1	14	18	4
*Staphylococcus epidermidis*	4	3		4	5	
*Staphylococcus aureus*	4	4		3	4	
*Streptococcus pharyngitis*	3	3		3	3	
Others	2	4		4	6	
Fungi (*Candida albicans*)	2	2	0	2	2	0
Total			4			11

## 4. Discussion

The term “horseshoe anal fistula” refers to a specific type of complex anal fistula characterized by its circular or horseshoe shape. This condition is typically identified by the presence of 2 or more external openings around the anus, with several branches extending on either side of the anal region. The designation “horseshoe” derives from the intricate pattern and shape reminiscent of a horseshoe.^[17,18]^ Horseshoe anal fistulas are predominantly located in a superior position relative to the sphincter, often towards the posterior aspect of the anal canal.^[19]^ It is clinically observed that a perianal abscess may spread to the sciatic fossa on both sides, with the infection extending to the deep space behind the anal canal. Surgical intervention is considered the primary treatment modality for this condition.^[20]^

AID is a common operation method for anal fistula, but the high posterior horseshoe anal fistula has special anatomical position, complex shape, deep wound, and large damage. If the cavity cannot be completely eliminated during suture, there is also a risk of recurrence and infection.^[21–23]^ Therefore, it is very important to improve the traditional surgical method and find a surgical method with unobstructed drainage and minimal trauma. Radial incision combined with tunnel floating line drainage is a method combining radial incision of sphincter with counterpart drainage, which has the advantages of less trauma and high radical cure rate.^[24]^ Previous study^[25]^ has confirmed that this operation is safe and effective, and can improve the one-time radical cure rate of anal fistula. The comparison of this study showed that the operation time, intraoperative bleeding, primary cure rate, wound healing time and postoperative 6 VAS score of the joint group were better than those of the control group, it is suggested that radial incision combined with tunnel floating line drainage is a highly practical and effective surgical method, which can improve the clinical efficacy, reduce the pain of patients, and promote wound recovery. In this study, the 2 surgical methods both adopt thread hanging treatment, the difference is that the internal opening is curved incision or radially incised. Relatively speaking, the radial incision is easier to operate, with sufficient drainage, less pain for patients, and can shorten the wound healing time. Other study^[4]^ has shown that high horseshoe anal fistula has a wide range of lesions, complex fistula shape, multiple branches, and many postoperative complications, which is a thorny problem for clinicians. Radial incision has less damage and scar to perianal and sphincter, which can reduce the probability of infection and the risk of anal deformity, so there are fewer complications.^[26,27]^

The goals of anal fistula treatment include full removal of fistula, maximum retention of anal function, and reduction of wound healing time.^[28]^ It has been reported^[29]^ that some patients after anal fistula surgery will show decreased anal function, change of stool and urine and living habits, which is closely related to sphincter injury and long-term nonunion of anal fistula. In this study, 1 month after operation, Wexner anal incontinence score in the combined group was significantly lower than that in the control group, indicating that radial incision combined with tunnel floating line drainage could better protect the anal function of patients. This is because the radial incision has less damage to the sphincter and less impact on anal function. At the same time, there was no difference in the 6-month recurrence rate between the 2 groups in this study, indicating that this operation can fully drain without increasing the postoperative recurrence rate. The following points should be noted during the operation: (1) strengthened preoperative exploration and determined the position of the internal mouth. (2) Effectively and thoroughly removed the fistula and the decayed tissue in the cavity, and repeatedly flushed to avoid leaving a dead cavity. (3) Selected the number of radial incisions according to the length of fistula to reduce tissue damage. (4) Paid close attention to the patency of drainage to ensure adequate drainage.

Perianal suppurative infection is the main cause of anal fistula. Under normal circumstances, the perianal area of human body is dominated by nonpathogenic bacteria, and the microbial flora is in balance. Under pathological conditions (such as anal fistula, hemorrhoids, etc), the balance of perianal flora is disordered, which leads to the decline of immune function and affects postoperative recovery.^[30]^ In addition, anal fistula surgery is traumatic and polluting, which will not only lead to the destruction of rectal mucosal barrier function, but also lead to bacterial infection.^[31]^ A study that conducted by Hezhai Yin et al^[32]^ has shown that the number of perianal bacteria strains in patients with perianal diseases after surgery is higher than that before surgery, and the surgical method will also have an impact on the number of new strains. The results of this study showed that the new increment of perianal bacteria in the joint group was notably less than that in the control group on the first day after operation, which also indicates that the radial incision combined with tunnel floating line drainage has less trauma and less risk of postoperative infection. At the same time, this study found that the main pathogenic bacteria around the anus after surgery were gram-negative bacteria, including *E coli* and *K pneumoniae*, which should be actively treated with anti-infection.

## 5. Conclusion

In conclusion, the combination of RCTD is an effective method for treating high posterior horseshoe anal fistula. It is not only simple and easy to operate, but also can improve the cure rate, reduce the incidence of complications, and reduce the number of new bacteria around the anus, which is worthy of promotion and application. However, the number of cases in this study is small, the observation time is short, and the operation process still has room for optimization.

## Author contributions

**Conceptualization:** Zhengqing Yan.

**Data curation:** Hang Yi.

**Formal analysis:** Yong Zheng.

**Methodology:** Hang Yi.

**Supervision:** Zhengqing Yan.

**Writing – original draft:** Hang Yi, Yong Zheng, Zhengqing Yan.

**Writing – review & editing:** Yong Zheng, Zhengqing Yan.
